# Catch a Sperm! An Integrated Forensic Workflow Combining STK Sperm Tracker™, AmylasePaper and Dual PSA/α-Amylase Lateral Flow Testing

**DOI:** 10.3390/genes17101269

**Published:** 2026-10-13

**Authors:** Mayaas Hassan, Lucía Júlvez Martínez, Polina Khalizov, Jennifer Caroline Santos, Gabriela Roca, Sara C. Zapico

**Affiliations:** 1 Department of Chemistry and Environmental Sciences, New Jersey Institute of Technology, 161 Warren Street, Tiernan Hall 365, Newark, NJ 07102, USA; mayaaskhassan@gmail.com (M.H.);; 2 SERATEC^®^, Gesellschaft für Biotechnologie mbH, Ernst-Ruhstrat-Strasse 5, 37079 Göttingen, Germany; 3 Anthropology Department and Laboratories of Analytical Biology, National Museum of Natural History, Smithsonian Institution, 10th and Constitution Ave, NW, P.O. Box 37012, Washington, DC 20560, USA

**Keywords:** semen, saliva, semen–saliva mixtures, STK Sperm Tracker, AmylasePaper, PAM test

## Abstract

**Background/Objectives**: Body fluid identification is essential in sexual assault investigations because it supports crime scene reconstruction and guides DNA analysis. However, biological traces are often limited, so it is necessary to maximize information from a single sample while minimizing consumption. Evaluation of body fluids by methods of different nature could enhance this detection without losing evidence. This study assessed the analytical compatibility of an integrated forensic workflow combining stain localization, body fluid identification, and DNA profiling to detect semen and saliva on different substrates. **Methods**: Semen, saliva, and semen–saliva mixtures were deposited on non-porous substrates (plastic, glass, and leaves) and porous substrates (cotton, denim, and leather). Semen stains were located using STK Spray^®^ Sperm Tracker™ or STK Lab^®^ Sperm Tracker™, while saliva was mapped using AmylasePaper. Samples were subsequently tested with the SERATEC^®^ PAM test for simultaneous detection of prostate-specific antigen (PSA) and α-amylase. DNA was extracted and quantified, followed by short tandem repeat (STR) profiling. **Results**: Sperm Tracker successfully located semen stains on all tested substrates, including stains aged for five days, while AmylasePaper detected saliva stains without observable interference from semen under the tested conditions. Neither mapping procedure interfered with the other or with subsequent PAM testing. PAM test correctly identified semen and saliva individually and in mixtures after all mapping steps. DNA was successfully recovered following the complete workflow, and full STR profiles were obtained from all non-porous samples and from porous samples with sufficient DNA quantities. **Conclusions**: The proposed workflow enables sequential localization, protein-based identification, and genetic characterization of semen and saliva while preserving evidentiary value. These findings support its application in sexual assault investigations to maximize biological information recovery while reducing sample consumption.

## 1. Introduction

Body Fluid Identification (BFID) can assist investigators in reconstructing the sequence of events at crime scenes [1,2]. In sexual assault cases, the detection of semen, blood, or saliva could lead, in many cases, through DNA analysis, to the identification of the perpetrator and the victim [3]. However, biological evidence is not always possible to recover from the victim’s body, making it necessary to search for these body fluids at crime scenes, in bedclothes, clothing, and objects [4,5].

One of the approaches to locate these fluids is the use of Alternative Light Sources (ALS), based on the photoluminescent properties of the fluids [6]. However, it is not specific to a body fluid, and it could produce false positives with many commercial products [7]. Seminal acid phosphatase (SAP) is secreted in high concentration by the prostate gland, and it could be identified based on its enzymatic activity, producing a change in the color of the substrate if semen is present [3,4]. It also produces false positives with other biological fluids and plant-derived substances [3,8]. A few years ago, STK Spray^®^ Sperm Tracker™ and STK Lab^®^ Sperm Tracker™ were introduced as non-toxic presumptive tests for the detection of SAP in objects and fabrics. It works through the application of a spray or by pressing a paper covered with the substrate, respectively, and observing the fluorescent signal under 365/366 nm UV light [9,10]. According to previous studies, both versions of the Sperm Tracker™ present high specificity and sensitivity for the detection of human semen, even with old stains [9,10,11]. The Spray version could also be applied directly at the crime scene as a first-pass screening tool [3].

Saliva also has a method to map its stains in fabrics, the Phadebas^®^ test or AmylasePaper from SERATEC^®^, both of which are based on the capacity of the α-amylase from the saliva to hydrolyze the starch [12,13,14] and the ability to locate the fluid, though with variability among textiles [13].

The next step could be the detection of body fluid through the identification of a specific protein. For this purpose, one of the most common approaches is the use of lateral flow immunochromatographic (LFI) tests [15], similar to COVID-19 tests. They are based on antigen–antibody reactions and developed against a specific protein in the body fluids: for blood, hemoglobin or glycophorin; for saliva, α-amylase; for semen, prostate-specific antigen (PSA) or semenogelin; for menstrual blood, D-dimer [16].

LFI tests enhance the identification of body fluids and can be performed at the crime scene. Despite advances in forensic semen identification, the performance of detection methods may vary depending on the substrate and environmental conditions [17]. Consequently, the ability to locate latent biological traces remains critical for their successful recovery and subsequent DNA analysis. While previous studies have demonstrated this, standardized comparative investigations evaluating standard and multiple biological sample types under identical experimental conditions are still limited [17].

Another important consideration in the examination of suspected body fluid stains is the potential consumption of limited sample material during testing. Laboratory workflows differ in how body fluid identification is integrated into casework: depending on the laboratory’s procedures, case circumstances, and the amount of material available, samples may undergo a single identification test or proceed directly to DNA extraction [18,19]. While direct DNA analysis may conserve material otherwise required for multiple screening tests, it can involve unnecessary expenditure of time and resources if the sample is unsuitable or not relevant to the case and does not, by itself, establish the biological source of the recovered DNA. Protein-based tests can support the targeted identification of body fluids and may also be applied at the crime scene. However, their findings generally remain presumptive and must be interpreted in the context of the sample origin, case circumstances, and known limitations of the test [20].

At the moment, it is imperative to find approaches that prioritize the retrieval of biological information from the sample and the possibility of generating a DNA profile [21]. LFI tests present this option. Semen and saliva are the most common encounter fluids in sexual assault cases. SERATEC^®^ has recently developed a new LFI, PAM test, for simultaneous detection of PSA and α-amylase [22].

An additional challenge in the development of integrated forensic workflows is to understand the biochemical compatibility between sequential analytical procedures. Stain-mapping methods, protein-based identification assays (LFI), and DNA profiling rely on fundamentally different molecular principles, including enzymatic reactions, antigen–antibody recognition, nucleic acid extraction, and PCR amplification. Consequently, reagents or treatments applied during an early screening step may potentially alter target proteins, affect subsequent immunochromatographic interactions, compromise DNA integrity, or introduce substances that interfere with downstream amplification.

Taking into account the techniques, methodologies, or analytical tools available for BFID, the main aim of this study was to answer the aforementioned question by evaluating the efficiency of a combined forensic workflow consisting of stain mapping, protein-based body fluid identification, and DNA profiling for the detection of semen and saliva deposited on different substrates. As a result, it was necessary first to test the performance of Sperm Tracker™ and AmylasePaper as mapping techniques, after that PAM test to identify PSA and α-amylase proteins as seminal fluid and saliva indicators, and finally STR profile quality analyses after these treatments.

This article experimentally evaluated a forensic workflow beyond the individual analytical performance of each method, and if it is influenced not only by the biological fluid involved (semen or saliva), but also by the matrix (substrate) in which the stain is deposited. The study assessed whether biological evidence retains its evidential value throughout a sequential analytical process in porous and non-porous materials. It also determined whether sufficient genetic material can be preserved and recovered for subsequent DNA profiling.

## 2. Materials and Methods

**Figure 1:**
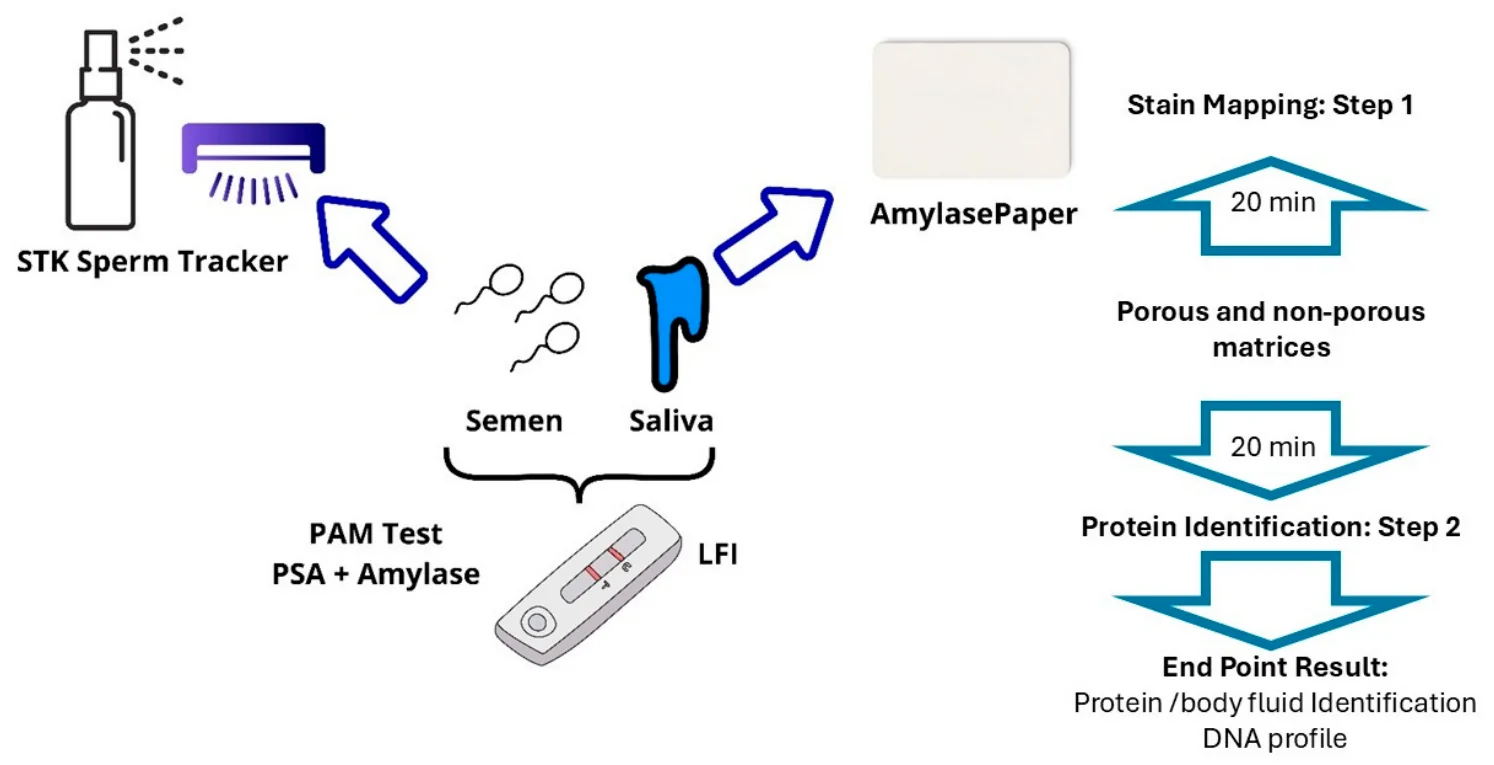
General Experimental Design and Testing. Designed with Canva online platform and PowerPoint.

**Figure 2:**
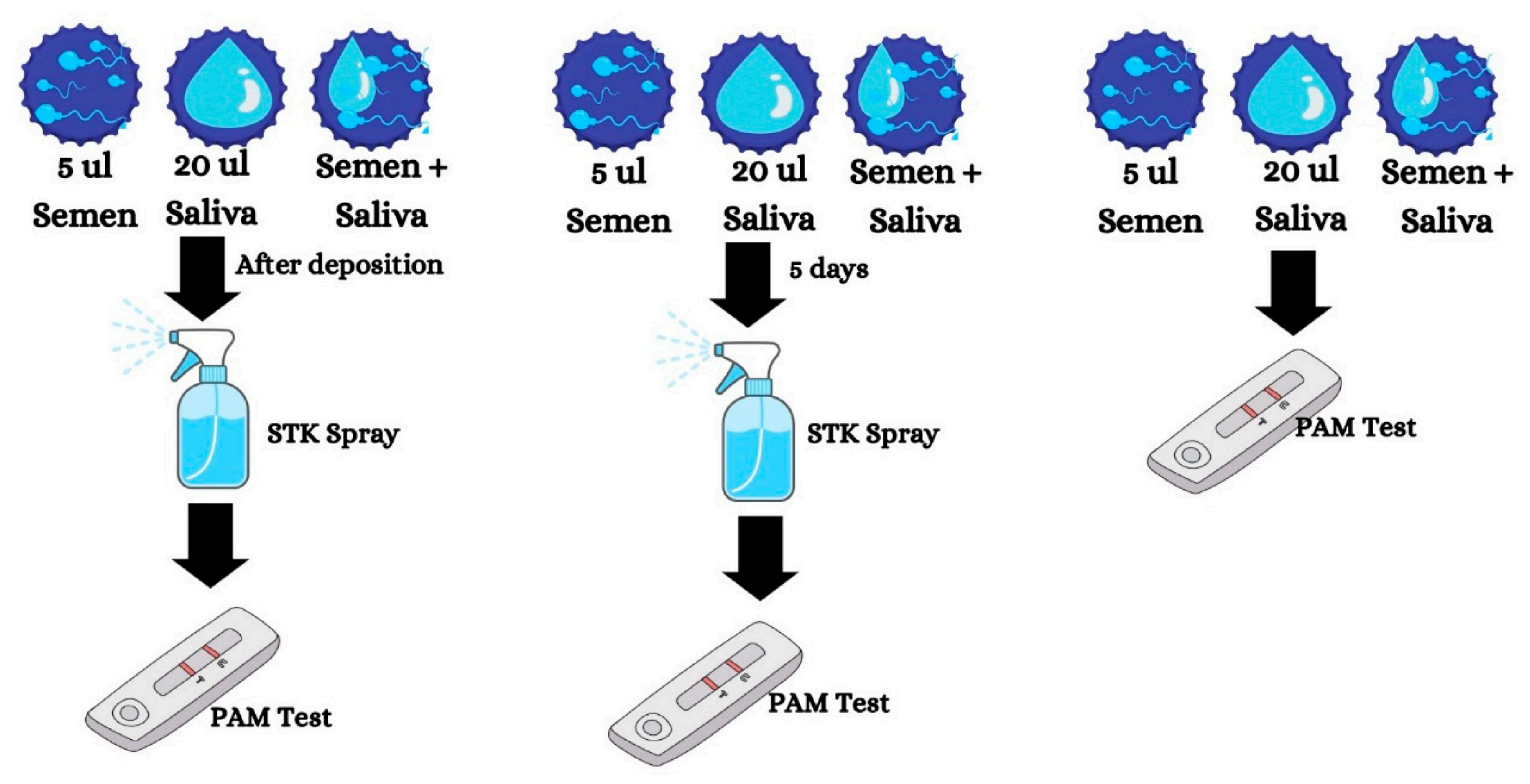
Experimental setup of the objects showing plastic trays. In Experiment 1, STK Spray was applied just after deposition of the stains. In Experiment 2, STK Spray was applied five days after deposition of the stain. Stains were also assessed with PAM test alone as a control. Glass and Leaves were also tested. Designed with Canva online platform.

**Figure 3:**
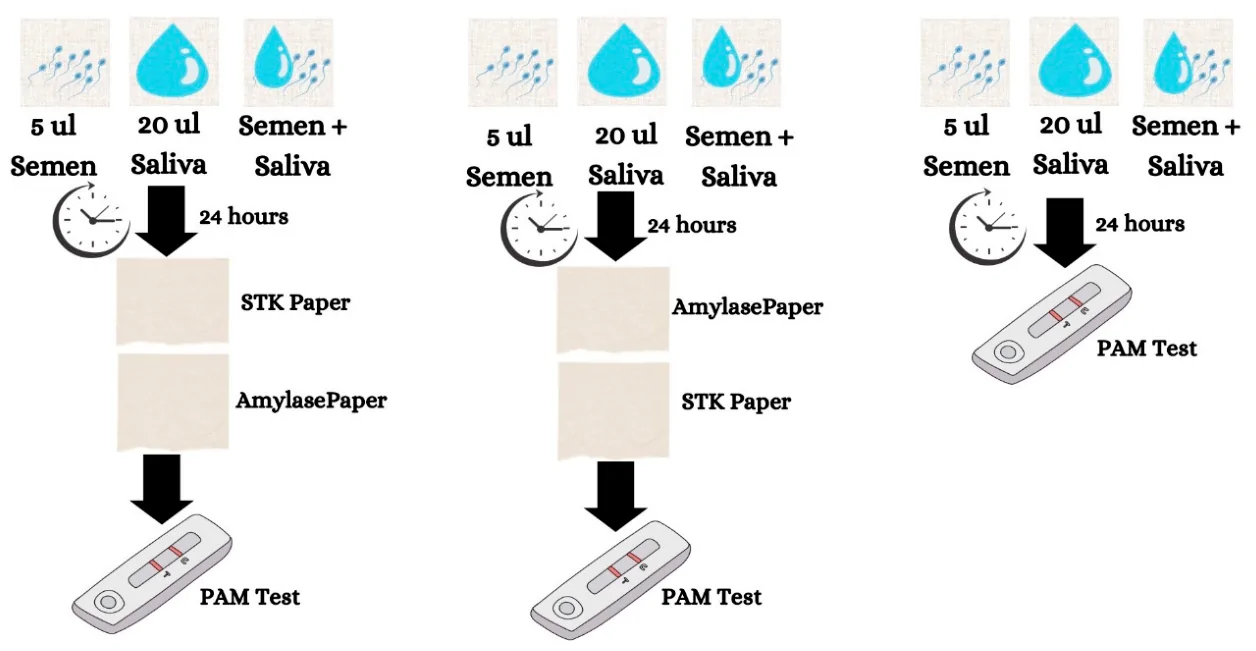
Experimental setup of the fabrics showing Cotton White. In Experiment 1, after 24 h of stain deposition, STK Paper was applied followed by AmylasePaper. In Experiment 2, after 24 h of stain deposition, AmylasPaper was applied followed by STK Paper. After mapping, in both experiments, PAM test was used to detect PSA and α-amylase. An additional control experiment was done, testing PAM alone 24 h after stain deposition. Cotton Black, Denim White, Denim Black, and Leather were also tested. Designed with Canva online platform.

General experimental design and testing are depicted in Figure 1. Specific workflows for non-porous and porous surfaces are displayed in Figure 2 and Figure 3.

Two workflows were assessed:

Workflow I: simplified compatibility model: STK → PAM test → DNA

Workflow II: expanded dual-fluid mapping model: STK ↔ AmylasePaper → PAM test → DNA

Together, these two experimental designs allowed the integrated workflow to evaluate a simpler STK–PAM interaction model on non-porous surfaces and a more complex dual-mapping strategy on porous substrates.

1. Biological sample

A vial of bulk semen from Caucasian males was purchased from Lee Biosolutions (Maryland Heights, MO, USA). Saliva was retrieved from a single female donor by spitting into an Eppendorf tube. The New Jersey Institute of Technology Institutional Review Board (IRB) approved the procedures related to human body fluid experimentation (protocol number: 2110013076). A total of 5 µL of Semen and 20 µL of Saliva were used for sampling, deposited alone and in combination on different substrates, with three repetitions (Figure 2 and Figure 3).

2. Substrates and stain preparation

*(a)*  *Non-porous surfaces Experimental setup: Objects*

A depiction of this experimental setup is shown in Figure 2.

Microscope slides, made of glass, 50 mL Falcon^®^ tubes caps, made of polyethylene (plastic), and leaves were used. Three replicates were conducted per fluid, combination of fluids, object, time (just after deposition and five days after deposition), and control of PAM testing alone to assess potential inhibition.

*(b)*  *Porous surfaces experimental setup: Fabrics*

A depiction of this experimental setup is shown in Figure 3.

Five fabrics: Cotton colors white and black; Denim colors white and black; and leather. Three replicates were conducted per fluid, combination of fluids, fabric, order of the screening (STK-AmylasePaper; AmylasePaper-STK), and control of PAM testing alone to assess potential inhibition.

*(c)*  *Environmental conditions and stain aging*

The samples were left under a Biosafety Cabinet Class II A2 model 1353 (Thermo Fisher Scientific, Waltham, MA, USA) (20 °C), between 30% and 50% humidity, and under a constant airflow at 973 m^3^/h. Time: zero (just after deposition) and 5 days for non-porous substrate, and 24 h after deposition for porous substrate. These times were selected based on the timing of discovering a crime.

3. Stain-mapping procedures

*(a)*  *STK Sperm Tracker™*

Sperm Mapping just after deposition and five days after deposition: STK Spray^®^ Sperm Tracker™ (AXO Science, Villeurbane, France) was spread on the objects and visualized with STK UV light (AXO Science, Villeurbane, France) after 1 min of incubation. In porous substrates 24 h after deposition, STK Lab^®^ Sperm Tracker™ (AXO Science, Villeurbane, France) was pressed on the fabrics for three minutes, following the manufacturer’s protocol, and visualized with STK UV light (AXO Science, Villeurbane, France). A picture was taken of the result in both cases.

*(b)*  *AmylasePaper*

On porous surfaces before or after STK Lab, AmylasePaper (SERATEC^®^, Göttingen, Germany) was applied according to the manufacturer’s instructions, taking a picture of the saliva stains.

*(c)*  *LFI test*

After mapping in both workflows, a cotton swab (cotton-tipped applicator, sterile, wood shaft; SARSTEDT, Nümbrecht, Germany) was moistened with SERATEC buffer (SERATEC^®^, Göttingen, Germany). Samples were collected by rubbing the swab on the fluid in a circular motion for ∼30 s. Then, swabs were incubated under agitation in 300 μL of SERATEC buffer for 10 min as recommended by the test manufacturer. Afterward, three drops (∼120 μL) of the resulting solution were added to the immunochromatographic tests (PAM test) using the plastic pipette provided with each test, and the results were recorded.

4. DNA isolation

Total DNA was isolated from one of the swab samples from each group in the LFI tests (no repetitions), applying a modification of the DNeasy Blood and Tissue Kit (Qiagen, Venlo, The Netherlands), according to our previously published protocol [15]. Briefly, as part of the buffer volume from the previous test was used during the test performance, an additional volume of SERATEC^®^ extraction buffer was added to the Eppendorf tube, up to 400 μL. While the swab was still inside the Eppendorf tube, 20 μL of proteinase K and 400 μL of AL buffer were added. The resulting mixture was vortexed for 15 s and incubated under agitation at 56 °C for 10 min. Then, 400 μL of ethanol was added and vortexed. Finally, the mixture was transferred to the column following the manufacturer’s protocol. The DNA was eluted in the final volume of 50 μL of AE buffer.

5. Total and human DNA quantification

Human DNA quantification was carried out using the PowerQuant System (Promega Corporation, Madison, WI, USA) and QuantStudio (Thermo Fisher Scientific, Waltham, MA, USA) following the manufacturer’s protocol and according to FBI and international guidelines [23].

6. STR profiling

The Promega PowerPlex^®^ Fusion 6C System (Promega Corporation) was used to amplify and characterize 23 autosomal STRs, 3 Y–STRs, and the Amelogenin gene according to the manufacturer’s protocol in 1 ng of DNA both for positive control and samples. Fragment analysis was carried out on SeqStudio (Thermo Fisher Scientific, Waltham, MA, USA). The fragment analysis was performed under the following parameters: 7 s injection time; 1200 V injection voltage; 1440 s run time; and 9000 V run voltage. DNA profiling was achieved through the Microsatellite Analysis software on Thermo Fisher Cloud (https://www.thermofisher.com/rs/en/home/digital-science/thermo-fisher-connect.html), accessed on 18 December 2025. A threshold of 150 RFUs was used for peak detection analysis. Quality of the DNA profile was only visually assessed due to the bulk semen DNA and the semen + saliva mixtures. The experiment was designed like this to obtain more information related to the STR profile variation in the whole process and not to identify an individual.

## 3. Results

1. Current workflow tested

Table 1 summarizes the current evidence on forensic workflows for semen and saliva analyses, encompassing stain localization, protein-based body fluid identification, and downstream DNA profiling. Our analysis highlights key considerations related to the sampling substrate, recovery methodology, and the integration of complementary data for evidence interpretation. Statistical analyses of the mean and error were not performed because the results were assessed as binary outcomes: present or absent. Therefore, all results classified as positive were considered 100% positive.

2. Mapping, environmental conditions and stain aging

*(a)*  *Non-porous Surfaces*

**Figure 4:**
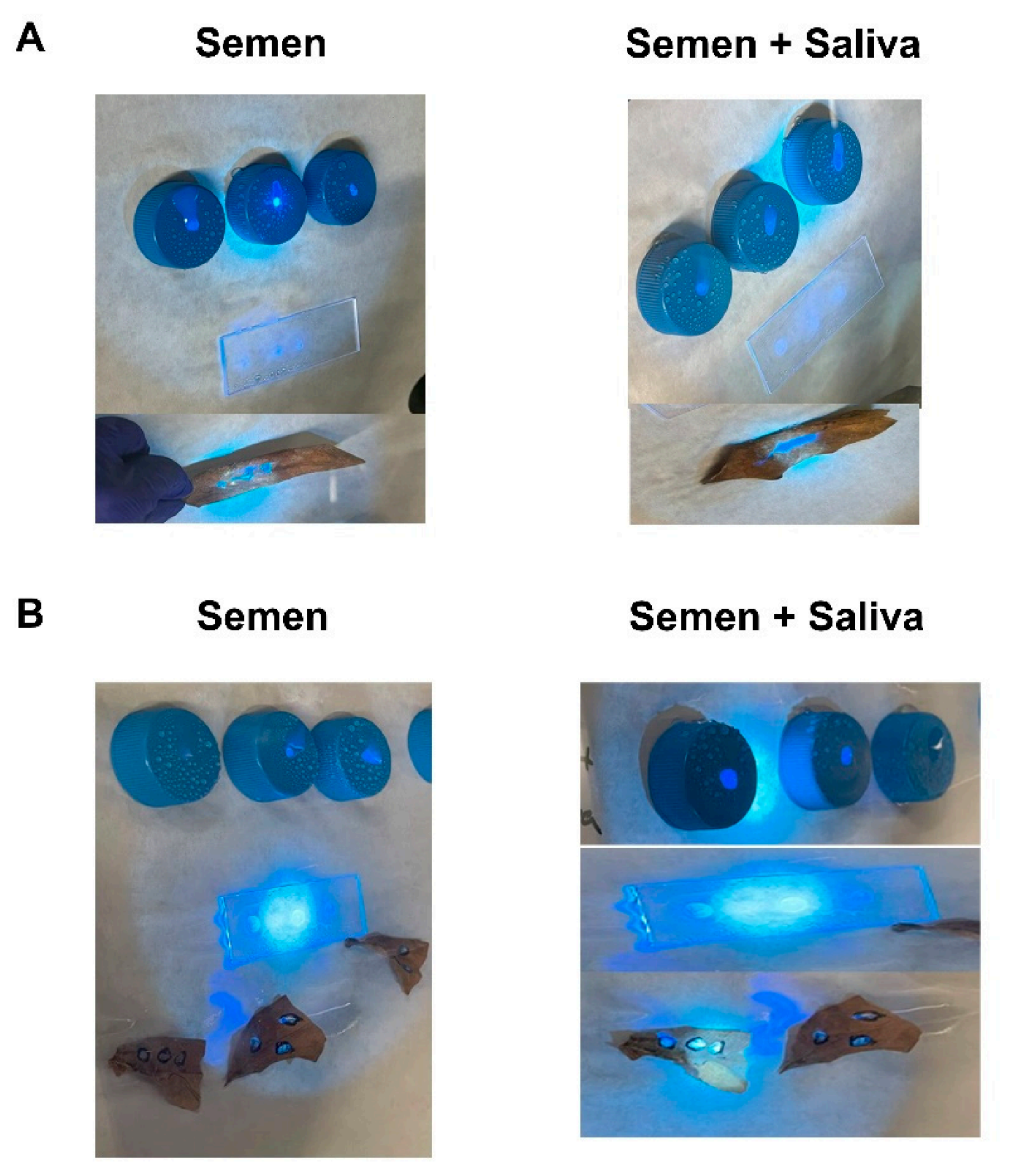
Seminal fluid localization in the three objects alone and in combination with saliva just after deposition (**A**) and five days after deposition (**B**).

On non-porous surfaces, localization and mapping with STK Spray^®^ Sperm Tracker™ enabled the successful detection of semen, both alone and in combination with saliva, across all tested substrates (plastic, glass, and leaves) immediately following deposition. Detection remained unaffected by environmental conditions, drying, or the post-deposition interval, as demonstrated in Figure 4.

*(b)*  *Porous Surfaces*

**Figure 5:**
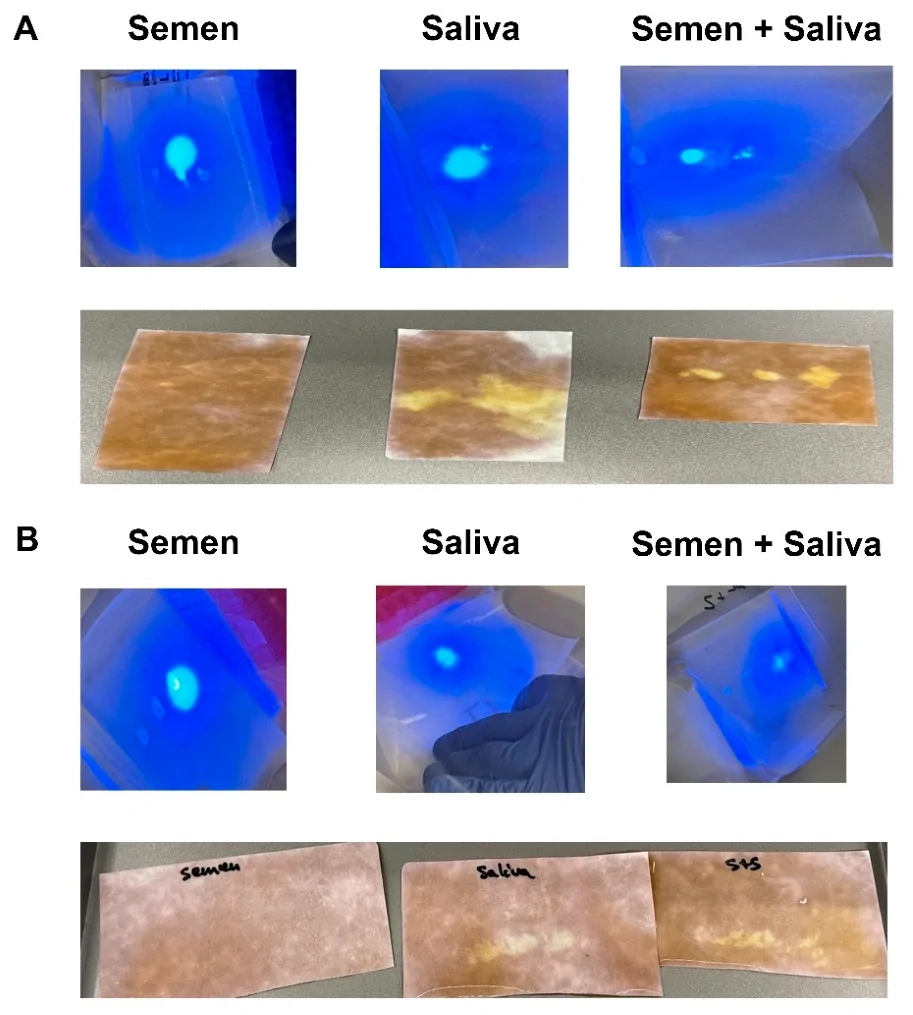
Detection of semen and saliva when Sperm Tracker is applied first (**A**) or AmylasePaper is applied first (**B**).

Semen stains were successfully localized on all five fabric types using STK Lab, with no evidence of cross-reactivity with saliva. Subsequent application of AmylasePaper enabled accurate saliva stain localization without interference from the previous STK Lab treatment. Importantly, reversing the analytical sequence yielded equivalent results, with AmylasePaper first detecting saliva stains and STK Lab subsequently identifying semen stains without mutual interference. These results are shown in Figure 5.

3. Protein detection by LFI tests

*(a)*  *Seminal fluid and saliva detection on non-porous surfaces*

**Figure 6:**
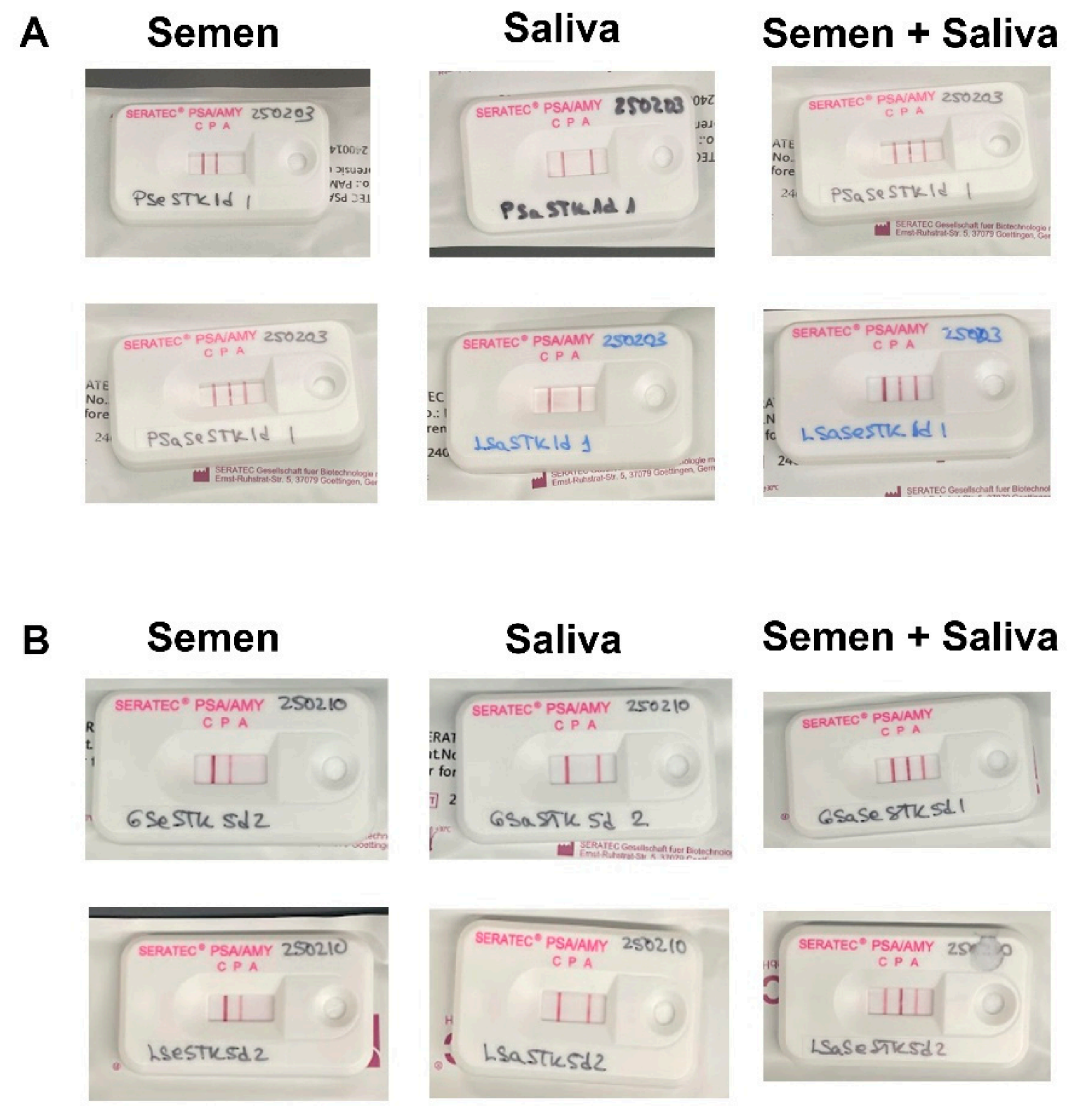
PSA and α-amylase detection alone and in combination just after deposition, showing plastic (**top**) and leaves (**bottom**) (**A**) or after 5 days post-deposition, showing glass (**top**) and leaves (**bottom**) (**B**).

Following semen stain localization with STK Spray^®^ Sperm Tracker™, PAM successfully and simultaneously detected PSA and α-amylase both immediately after deposition and after 5 days of aging. No interference between the detection methods was observed, demonstrating their compatibility within a sequential workflow. The results are shown in Figure 6.

*(b)*  *Seminal fluid and saliva detection on porous surfaces*

**Figure 7:**
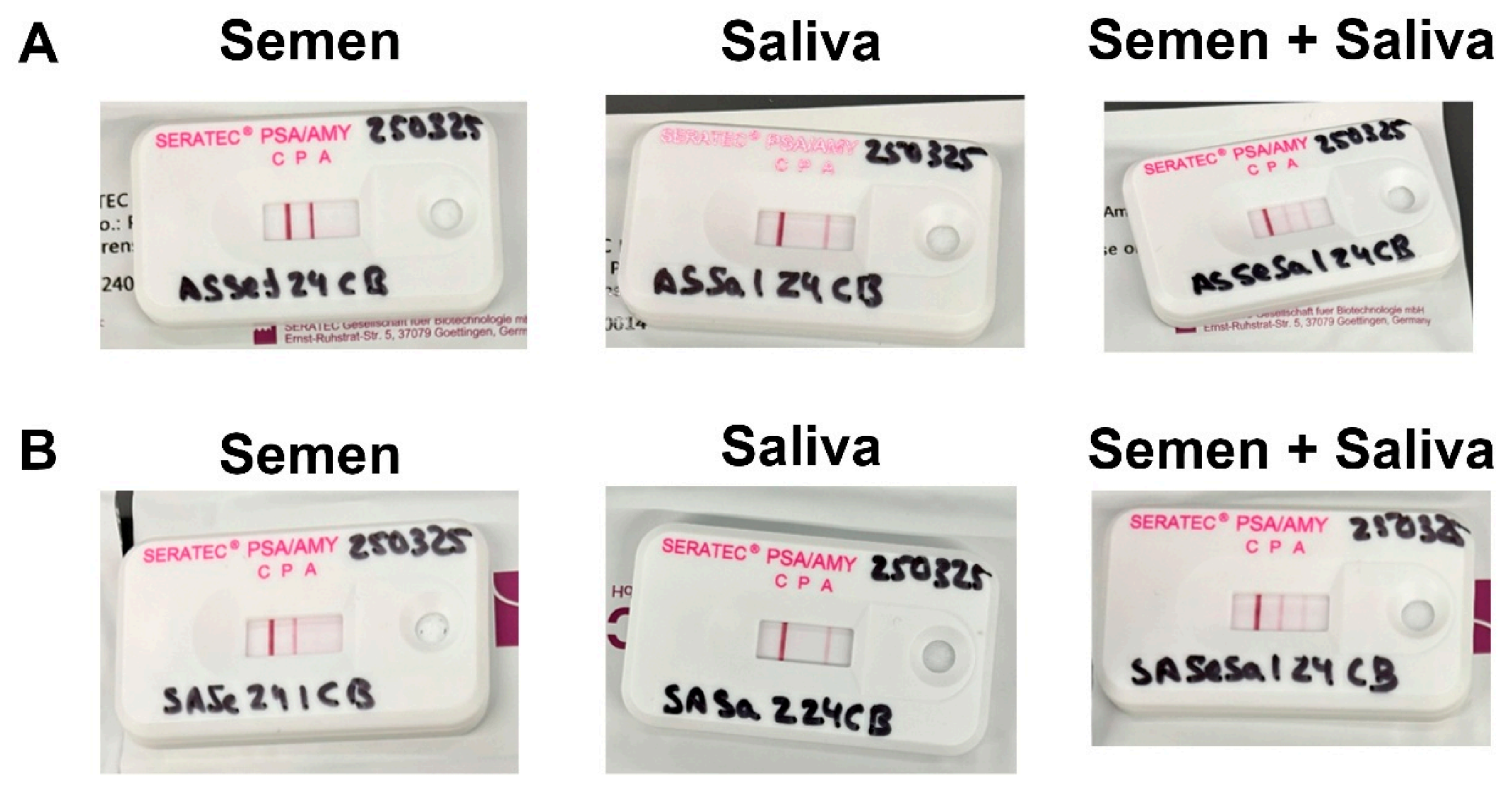
PAM test simultaneously detects PSA and α-amylase providing protein-based evidence consistent with the presence of seminal fluid and saliva in Cotton Black. (**A**) AS, AmylasePaper first, then STK; (**B**) SA, STK first, then AmylasePaper.

After sequential localization of semen and saliva stains using STK Lab^®^ Sperm Tracker™ and AmylasePaper, the PAM test successfully identified PSA and α-amylase, with results consistent with the expected body fluid composition. No effect of the application order was observed, demonstrating the compatibility of these techniques within a single forensic workflow. These findings are depicted in Figure 7.

4. DNA isolation and quantification

*(a)*  *Non-Porous Surfaces*

DNA was successfully isolated from the swabs after body fluid identification from non-porous surfaces in each experimental group. DNA concentrations obtained from the objects were adequate to perform the next step of profiling (between 0.1 and 9.4 ng/µL), with low degradation ratios, no inhibitors, and correctly identified female and male donors, as well as mixtures (Table 2).

*(b)*  *Porous Surfaces*

DNA recovery from the fabric samples was variable, with concentrations ranging from 0.0062 to 14.2 ng/µL. While most samples yielded less than 0.25 ng/µL, and some therefore failed to meet the minimum DNA input requirement for STR profiling (0.5 ng total DNA), no inhibition was detected and only moderate degradation was observed in a subset of samples. For samples generating complete profiles, male and female donors were accurately identified, including mixed contributors (Table 3).

5. DNA profiling

*(a)*  *Non-Porous Surfaces*

**Figure 8:**
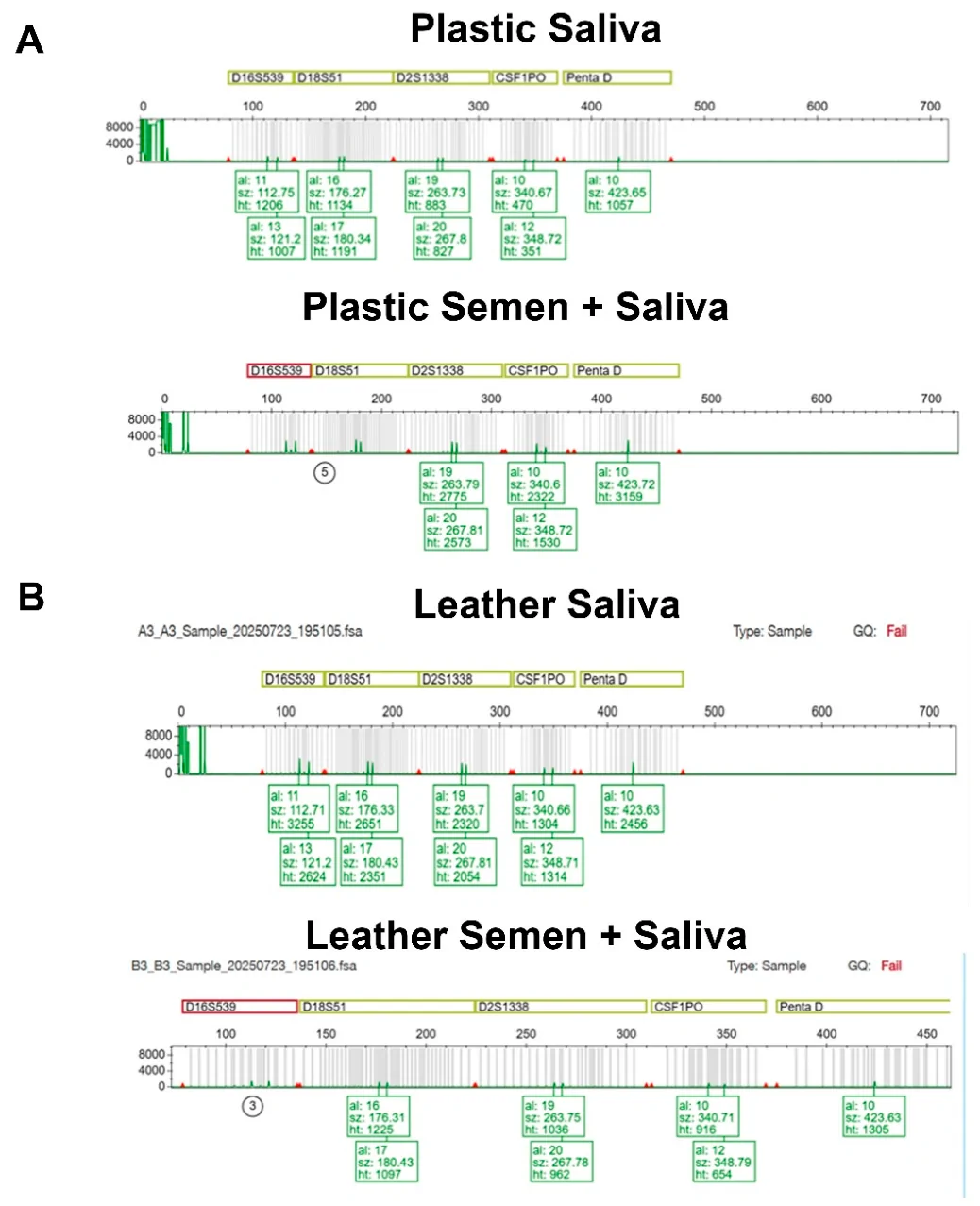
(**A**) Yellow channel of the STR analysis for the Saliva sample and Semen and Saliva samples deposited in plastic. (**B**) Yellow channel of the STR analysis for the Saliva sample and Semen and Saliva samples deposited in leather.

Complete DNA profiles were obtained from all DNA extracted from plastic and leaf samples per experimental group. Despite variation in signal intensity, as reflected by differences in peak height, mixed DNA profiles were successfully identified. Figure 8A presents a representative electropherogram channel from the saliva and semen + saliva sample deposited on plastic.

*(b)*  *Porous Surfaces*

Complete DNA profiles were obtained from leather samples per experimental group, as they contained sufficient DNA for STR amplification (minimum input: 0.5 ng total DNA). As observed, peak heights varied among samples; however, mixed DNA profiles were successfully detected. Figure 8B presents a representative electropherogram channel from the saliva and semen + saliva sample deposited on leather.

## 4. Discussion

The ultimate goal of forensic investigations is the identification of perpetrators and, in some cases, victims. While previous studies have explored the integration of body fluid identification and DNA analysis using a variety of molecular approaches [24,25,26,27,28,29], a key strength of the present study is the evaluation of multiple porous and non-porous matrices under standardized conditions. This design enabled a comprehensive assessment of the compatibility of stain localization, protein-based body fluid identification, and DNA profiling across diverse forensic scenarios.

A rapid workflow capable of detecting body fluids at the crime scene or during laboratory examination, while preserving samples for subsequent DNA profiling, would be highly advantageous for forensic investigations. Under the conditions evaluated in this study, the proposed sequential approach showed strong analytical performance and compatibility among the different detection methods. Notably, neither protein-based body fluid identification nor downstream DNA profiling was compromised by stain drying, aging, or prior mapping procedures, supporting the applicability of this workflow to a range of forensic scenarios.

The present study demonstrated the effectiveness of STK Sperm Tracker^®^ for semen localization on both porous and non-porous surfaces, as well as the ability of AmylasePaper to map saliva stains on non-porous substrates. The presence of seminal fluid and saliva was subsequently confirmed using the PAM test, which simultaneously detects PSA and α-amylase, and DNA profiling was successfully achieved in most samples. The objective of this work was not to assess the individual performance characteristics of these techniques, as previous studies have already established the sensitivity and specificity of STK Sperm Tracker^®^ for semen detection on a variety of objects and fabrics [3,9,10,11]. Rather, this study evaluated their compatibility within a sequential forensic workflow. To better simulate sexual assault evidence, a small volume of semen (5 µL) was analyzed both alone and in combination with a larger volume of saliva (20 µL). Consistent with previous reports [3], saliva did not interfere with semen detection, and semen stains were successfully localized across all tested matrices, including dry leaves. These findings highlight the broad applicability of STK Sperm Tracker^®^ for the examination of diverse forensic evidence while preserving the ability to perform downstream body fluid identification and DNA profiling.

This work further evaluated the compatibility of saliva mapping and protein-based body fluid identification within a sequential forensic workflow. Saliva stains were successfully localized on multiple fabric types using AmylasePaper, both before and after the application of STK Sperm Tracker^®^. Previous studies investigating AmylasePaper and the Phadebas test have primarily focused on sensitivity, specificity, and potential textile-related variability [12,13,30,31]. In the present work, three fabric types, denim, cotton, and leather, were examined, and no differences in saliva detection were observed before or after STK Sperm Tracker^®^ application, even in samples containing both saliva and semen. These findings differ from previous reports suggesting reduced detection efficiency on certain textile substrates, such as cotton and polyester [13]. However, comparisons should be made cautiously, as our study employed a fixed volume of saliva (20 µL) rather than a dilution series. To our knowledge, this is the first study to evaluate the combined use of STK Sperm Tracker^®^ and AmylasePaper for the complementary detection of semen and saliva within the same workflow. The results demonstrate compatibility between the two approaches and an absence of observable interference under the tested conditions. Nevertheless, the present study was not designed as a comprehensive assessment of specificity, and the findings should be interpreted within the experimental conditions evaluated.

An additional level of body fluid confirmation was provided through the application of the PAM test, which simultaneously detects PSA and α-amylase. In forensic casework, biological stains are often limited in quantity, partially degraded, concealed, or subjected to previous environmental exposure. Consequently, investigators must frequently balance the need for body fluid identification against the preservation of biological material for DNA profiling. To address this challenge, several direct-PCR approaches have been developed to maximize DNA recovery while minimizing sample consumption [18,19,21,32,33,34,35,36]. However, the absence of body fluid identification may limit the contextual information available for reconstructing crime events [37]. Therefore, the development of BFID methods that preserve material for downstream DNA analysis remains an important objective [21].

The workflow evaluated in this study was designed to address this need by sequentially combining stain localization using STK Sperm Tracker^®^ and AmylasePaper with subsequent body fluid identification through the PAM test following a single sample collection [7,26,38,39,40]. Our results demonstrated that PAM performance was not affected by prior mapping procedures, successfully detecting semen and saliva, both individually and in combination, across all porous and non-porous matrices examined. These findings support the compatibility of PAM with upstream stain-localization techniques and its integration into a comprehensive forensic workflow.

Because relatively few studies have evaluated the PAM test, evidence from studies investigating PSA assays alone remains relevant for interpreting the PSA component of the test. For example, Bitner (2012) [41] reported intermittent PSA-positive reactions in samples associated with lubricated condoms. However, those experiments differed from the manufacturer’s recommended protocol through the use of larger extract volumes and the omission of sample dilutions. Furthermore, the use of unused condoms may not accurately reflect routine forensic casework, where lubricant transfer is likely reduced. Collectively, these observations suggest that specific matrices may influence PSA detection under certain analytical conditions, although the practical significance of these effects in standard forensic investigations remains uncertain.

Additional studies have highlighted the importance of interpreting PSA results within the context of the sample matrix and biological source. For example, PSA-positive results have been reported in the absence of detectable spermatozoa in certain postmortem samples [42], whereas PSA persistence appears to be limited in vaginal swabs collected from living individuals several days after exposure [38]. Together, these findings suggest that PSA detection may vary depending on the biological and environmental context of the sample.

Consequently, protein-based body fluid identification results should be interpreted alongside other available evidence. In the present study, stain localization, protein detection, and DNA profiling provided complementary information, emphasizing the value of a multimodal approach to forensic evidence interpretation. Accordingly, the absence of one form of evidence, such as a DNA profile, should not automatically negate the significance of other independent findings.

Beyond the performance of the individual assays, this study demonstrated the compatibility of a sequential workflow integrating stain localization, protein-based body fluid identification, and DNA profiling. Although the workflow combines enzymatic, immunological, and genetic analyses, no interference was observed between the different stages. Complete STR profiles were obtained from all non-porous surface samples tested, while DNA recovery from porous substrates was more variable but did not indicate incompatibility among the analytical steps [43,44]. These findings support the feasibility of applying multiple complementary forensic techniques to the same biological trace while preserving material for downstream analyses.

## 5. Conclusions

To our knowledge, this is the first study to evaluate an integrated workflow combining stain localization using STK Sperm Tracker^®^ and AmylasePaper with protein-based body fluid identification using the PAM test for the analysis of semen and saliva evidence. Under the conditions tested, semen and saliva were successfully detected both individually and in combination across a range of porous and non-porous substrates. Furthermore, the PAM test reliably identified PSA and α-amylase following stain localization, and downstream STR profiling was successfully performed when sufficient DNA was recovered.

Overall, our findings demonstrate the feasibility of integrating complementary stain-localization, protein-identification, and DNA-profiling methods within a single sequential workflow. STK Sperm Tracker^®^, AmylasePaper, and the PAM test were analytically compatible across the tested forensic matrices while preserving material for subsequent genetic analysis. This approach maximizes the information that can be obtained from limited biological traces and supports a more comprehensive interpretation of semen and saliva evidence in forensic casework.

## Figures and Tables

**Table 1:** Summary of the results in each step and workflow.

	Substrate	Mapping	PAM Test	Aging	DNA Extraction	DNA Concentration	STR	Analytical Compatibility
**Workflow I** **Non-porous substrates**	Plastic, glass and plant leaves	+	+	0 point and 5 days	+	0.17–9.4 ng/µL	+	+
**Workflow II** **Porous substrates**	Black and White Cotton, Black and White Denim, and Leather.	+	+	24 h	+	0.0062–14.2 ng/µL	+ When DNA concentration was optimal	+ substract effect

**Table 2:** DNA concentration in ng/µL, degradation index, and male/female ratio in Saliva, Semen, Saliva and Semen samples retrieved from Plastic, Glass, and Leaves. Since all samples did not show any inhibitors, IPC column is not included.

Sample ID	Concentration (ng/µL)	Degradation Ratio	Male/Female Ratio
**Plastic Semen STK**	0.16	1.7	1.1
**Plastic Saliva STK**	0.36	2.78	0
**Plastic Saliva–Semen STK**	4.15	1.48	8.17
**Glass Semen STK**	0.29	1.33	1.11
**Glass Saliva STK**	1.07	2.03	0
**Glass Saliva–Semen STK**	1.82	1	14.38
**Leaves Semen STK**	0.62	1.11	1.03
**Leaves Saliva STK**	0.51	1.71	0
**Leaves Saliva–Semen STK**	3.67	0.52	7.08
**Plastic Semen PAM**	1.02	1.48	1.19
**Plastic Saliva PAM**	0.25	1.91	0
**Plastic Saliva–Semen PAM**	9.42	1.25	5.93
**Glass Semen PAM**	1.09	1.26	1.16
**Glass Saliva PAM**	0.32	2.04	0
**Glass Saliva–Semen PAM**	4.08	1.37	4.64
**Leaves Semen PAM**	0.17	1.3	1.06
**Leaves Saliva PAM**	1.09	4.13	0
**Leaves Saliva–Semen PAM**	3.97	1.68	10.26

**Table 3:** DNA concentration in ng/µL, degradation index, and male/female ratio in Saliva, Semen, Saliva and Semen samples retrieved from Cotton White, Cotton Black, Denim White, Denim Black, and Leather, from STK-Amylase and Amylase-STK. Since all samples did not show any inhibitors, IPC column is not included.

Sample ID	Concentration (ng/µL)	Degradation Ratio	Male/Female Ratio
STK Amylase Semen Denim White	0.025	2.56	1.82
STK Amylase Saliva Denim White	1.030	2.09	No Y Cq
STK Amylase Semen–Saliva Denim White	0.021	1.88	13.5
Amylase STK Semen Denim White	0.042	1.62	1.4
Amylase STK Saliva Denim White	0.118	2.66	No Y Cq
Amylase STK Semen–Saliva Denim White	0.063	2.11	5.06
PAM Semen Denim White	0.073	1.67	1.3
PAM Saliva Denim White	1.165	2.47	No Y Cq
PAM Semen–Saliva Denim White	0.286	2.93	13.47
STK Amylase Semen–Saliva Denim Black	0.011	3.14	1.47
STK Amylase Saliva Denim Black	0.092	1.86	No Y Cq
STK Amylase Semen–Saliva Denim Black	0.097	1.22	1.83
Amylase STK Semen Denim Black	0.006	No Deg Cq	1.52
Amylase STK Saliva Denim Black	0.018	26.5	No Y Cq
Amylase STK Semen–Saliva Denim Black	0.015	1.81	2.54
PAM Semen Denim Black	0.025	6.83	1.28
PAM Saliva Denim Black	0.007	2.25	No Y Cq
PAM Semen–Saliva Denim Black	0.033	2.06	1.98
STK Amylase Semen Cotton White	0.027	1.55	1.33
STK Amylase Saliva Cotton White	0.021	1.89	No Y Cq
STK Amylase Semen–Saliva Cotton White	0.089	10.02	4.54
Amylase STK Semen Cotton White	0.013	1.29	1.25
Amylase STK Saliva Cotton White	0.024	2.04	No Y Cq
Amylase STK Semen–Saliva Cotton White	0.096	1.4	1.61
PAM Semen Cotton White	0.231	1.61	1.15
PAM Saliva Cotton White	0.502	1.31	No Y Cq
PAM Semen–Saliva Cotton White	1.492	1.45	12.49
STK Amylase Semen Cotton Black	0.014	1.34	2.01
STK Amylase Saliva Cotton Black	0.006	1	No Y Cq
STK Amylase Semen–Saliva Cotton Black	0.024	1.52	2.41
Amylase STK Semen Cotton Black	0.064	1.58	1.36
Amylase STK Saliva Cotton Black	14.263	2.04	No Y Cq
Amylase STK Semen–Saliva Cotton Black	0.221	2.38	11.17
PAM Semen Cotton Black	0.032	1.68	1.27
PAM Saliva Cotton Black	0.011	1.28	No Y Cq
PAM Semen–Saliva Cotton Black	0.023	2.21	1.91
STK Amylase Semen Leather	0.121	31.3	1.48
STK Amylase Saliva Leather	2.068	2.35	No Y Cq
STK Amylase Semen–Saliva Leather	1.293	1.66	39.79
Amylase STK Semen Leather	0.118	56.57	1.46
Amylase STK Saliva Leather	0.223	2.81	No Y Cq
Amylase STK Semen–Saliva Leather	1.008	2.11	57.93
PAM Semen Leather	0.235	1.87	1.22
PAM Saliva Leather	0.682	1.75	No Y Cq
PAM Semen–Saliva Leather	9.463	1.92	12.89

## Data Availability

Dataset available on request from the authors.
